# Physical activity and quality of life of patients with fibromyalgia

**DOI:** 10.17159/2078-516X/2023/v35i1a14781

**Published:** 2023-02-15

**Authors:** L Smith, M Croucamp

**Affiliations:** Department of Sport and Movement Studies in the Faculty of Health Sciences, University of Johannesburg, Doornfontein Campus, Johannesburg, South Africa

**Keywords:** Fibromyalgia Syndrome, FMS, exercise, fibromyalgia impact

## Abstract

**Background:**

Fibromyalgia Syndrome (FMS) has been linked to decreased social functioning, poor mental health, and quality of life (QOL). Increased physical functioning and activity can result in improvements in social, mental and overall health, as well as lowered depression and anxiety levels.

**Objectives:**

The aim of this study was to determine physical activity levels and QOL amongst patients diagnosed with fibromyalgia in the Johannesburg region of South Africa.

**Methods:**

The research design was cross-sectional. Descriptive and quantitative data were collected. FMS patients (n=38) completed an online questionnaire on the Google Forms platform. The questionnaire was comprised of four components, namely Demographics, the Global Physical Activity Questionnaire (GPAQ), the Fibromyalgia Impact Questionnaire (FIQR), and the Short Form-36 (SF-36). During data analysis, descriptive characteristics and correlations were computed. The significance level was set at p ≤ 0.05.

**Results:**

Results revealed high FIQR scores (67%) accompanied with low QOL scores (<50% in all domains). There was no correlation between physical activity and FIQR, and physical activity and QOL.

**Conclusion:**

High scores on the impact of FMS were associated with lower overall QOL scores. However, the relationship between physical activity, and the impact of FMS and QOL remain inconclusive.

Fibromyalgia Syndrome (FMS) is a chronic disorder characterised by musculoskeletal pain and a heightened response to the sensation of pressure.^[1,2]^ The condition is more prevalent in the adult female population and is influenced by socioeconomic and cultural factors.^[3]^ From a healthcare perspective, many patients report inconsistencies within the healthcare system and poorly coordinated care due to the complexity of the illness.^[4]^ In addition, healthcare practitioners are of the perception that FMS is not a real medical condition, which often results in debates and challenging encounters between healthcare practitioners and patients.^[4]^ An exploratory study reported a patient stating the following: ‘One day I walked into the [doctors’] rooms, [and] he told me once again ‘there’s nothing wrong with you’, and I told him ‘But doctor, this is impossible. I can’t sleep, I can’t turn, I can’t get up from the bed. I have difficulty driving’. ^[1]^ On the other hand, a large proportion of FMS patients in South Africa are disadvantaged by limited access to healthcare facilities, healthcare staff and treatment owing to the disadvantaged communities they reside in.^[1]^ Although a multidisciplinary and biopsychosocial approach to FMS has been advocated for, there is still a need to increase awareness.^[2]^ Of the numerous methods identified to manage the illness, such as relaxation exercise, social support, medication, and prayer, to name a few, exercise has been proven to be the most successful among FMS patients.^[5]^ Despite the efficacy of exercise in slowing down the progression of FMS and the management of its symptoms, many patients are still not engaging in regular physical activity.^[5]^

The effects of physical activity on FMS and overall quality of life (QOL) have been well-documented. Several studies comparing the effects of aerobic exercise and muscle strengthening programmes among patients with FMS have yielded benefits with fatigue, sleep, tender point count, pain, fitness and QOL.^[6]^ Interestingly, 12 weeks after exercise had been stopped among patients, those who had previously engaged in muscle-strengthening activities experienced a reoccurrence of symptoms, while the benefits of aerobic exercise lasted for a longer period of time.^[6]^ In terms of aerobic activities, aquatic exercise and land-based exercise showed similar improvements in the physical and mental health in this population; however, programmes that combine stretching, aerobic and muscle strengthening activities are the most effective in improving the QOL and the overall well-being of patients with FMS.^[6]^ Regardless of the debates relating to the most suitable mode or intensity of exercise for FMS patients, physical activity in any form has been shown to improve QOL in these patients.^[7]^ However, physical inactivity in South Africa is a major concern as the country has the highest prevalence of inactivity compared to other countries, such as developing countries like Poland and Romania.^[8]^ In conjunction with increasing physical activity, there is also a need to emphasise a decrease in sedentary time for optimal benefit.^[9]^ For this reason, the purpose of this research in determining physical activity levels and QOL amongst FMS patients in the Johannesburg region was established.

## Methods

### Study design and participants

This study used a cross-sectional research design. Descriptive and quantitative data were collected to achieve the aims and objectives.

A total of 38 participants from the Johannesburg region diagnosed with FMS formed the sample population of this pilot study. Participants of both genders who have been diagnosed with FMS by a qualified physician were recruited. The inclusion criteria were participants between the ages of 18 and 65 years diagnosed with FMS and who have access to the internet to complete the online questionnaire.

### Ethical considerations

This research was approved by the institution’s Research Ethics Committee (REC-229-2019). Principles of autonomy, beneficence, non-maleficence, and justice were applied and adhered to throughout the research process.

### Sampling and recruitment

Researchers contacted rheumatologists and general practitioners and requested referrals of their patients who have been diagnosed with FMS. Once participants had been contacted and agreed to participate in the research, they were asked to refer other qualifying patients by means of snowball sampling. All participants who met the inclusion criteria and completed the questionnaire formed part of the study’s sample.

### Data collection

The three questionnaires used in this study were collated and uploaded onto the Google Forms Platform (Appendix A). A link to the online questionnaire was distributed to the referred participants via email. Once accessed, the duration given to complete the questionnaire was 20 to 30 minutes.

### Measuring tools and instruments

The online version of this questionnaire was a combination of three questionnaires, namely, The Fibromyalgia Impact Questionnaire – Revised (FIQR), The SF-36, and The Global Physical Activity Questionnaire (GPAQ). In addition, a demographics section was included at the beginning of the questionnaire with the purpose of defining the characteristics of the sample population.

### Statistical analysis

Data were computed on the Statistical Package for Social Sciences (SPSS) for Windows version 15.0 (SPSS Inc., Chicago, IL, USA). Together with descriptive statistics, inferential statistics were also computed. For the purpose of determining normality, the Shapiro-Wilk test was given. Pearson’s Correlation Coefficient was used to establish the relationship between variables. The level of significance was set at 5% (p ≤ 0.05). The Bonferroni Adjustment was applied as a post hoc test and was set at p ≤ 0.0167. This was computed to account for the multiple comparisons between variables and to avoid false statistical inferences.^[10]^

## Results

The results presented in this section include the demographics of the sample, physical activity levels (assessed by the GPAQ), the impact of FMS on daily life (assessed by the FIQR), QOL, relationships between QOL and FMS impact, results of the GPAQ in relation to total time spent being physically active, total time spent being sedentary and relationships between active time, sedentary time, FMS impact and QOL.

### Sociodemographic aspects

Table 1 reflects the demographic data of the sample. Of the 38 participants who completed the online questionnaire, 37 were females (97%) and 1 was male (2.6%) (Table 1). The dominant age grouping for FMS in this sample was 41 to 50 years, as 32% of the participants were aged in this range. Only one participant (2.6%) was aged between 18 and 20 years. Majority of the participants (68%) were Caucasian, and no participants were of Black African descent. A large proportion of the sample (42%) indicated full-time employment, while 16% of the participants were unable to work.

### Physical activity

**Fig. 1:**
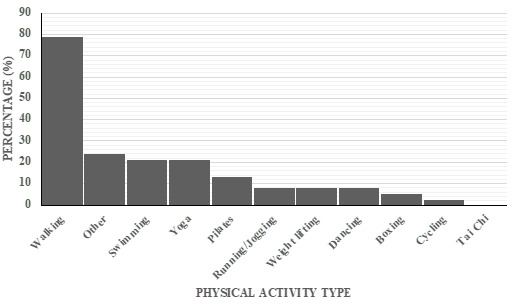
Various activities performed by participants (%) (n=31). Participants could select multiple responses.

The various activities performed by participants who engage in exercise are illustrated in Figure 1. A total of 31 participants (82%) indicated that they currently engage in physical activity (Figure 1). Participants could select multiple responses to this question, resulting in the total exceeding 100%. The most popular type of physical activity was walking (79%), while none of the participants indicated participation in Tai Chi.

Participants stated that they spend an average of 903 minutes (15.1 hours) a week engaged in some sort of physical activity. Total sedentary time on average was reported as 5.8 hours a week.

### The impact of FMS on daily life (FIQR)

The impact that FMS has on the daily life of patients with FMS is demonstrated in Table 2. Table 2 shows that FMS was shown to have a large impact on the participants’ daily lives, reflected as a mean total FIQR score of 67%. The higher the FIQR score, the larger the impact it has on the participant’s life.

### Quality of life (SF-36)

Table 3 reflects the QOL scores across eight domains. The scores for all QOL domains were below 50%, indicating a low score (Table 3). Role limitations due to physical health (15%), role limitations due to emotional health (22%) and energy/vitality (22%) exhibited the lowest scores, while social functioning yielded the highest mean score (40%), although it was also below 50%.

### The relationship between FIQR and SF-36

The correlations between FIQR and QOL are depicted in Table 4. Although all domains reflected a significant relation with QOL, the most significant correlation exists between FIQR and emotional wellbeing and the weakest correlation exists between the FIQR and role limitations due to emotional health (Table 4).

### Relationship between total time active, sedentary time and SF-36

Table 5 shows the correlations between total active time, sedentary time and QOL of the sample. Table 5 illustrates that neither of the domains in QOL showed a statistically significant correlation total active time and sedentary time.

## Discussion

This study aimed to determine the QOL amongst patients with FMS by defining physical activity levels and the impact of FMS.

### Demographic aspects

The majority of the sample consisted of females, namely 97%. Similarly, research shows that FMS is more prevalent in the female population, with a ratio of 6:1 to their male counterparts.^[3,11]^ A study assessing the QOL amongst FMS patients yielded similar findings, with a 95% prevalence of females in the sample.^[12]^ The majority of the sample (71%) were over the age of 41 years, which is line with what is contained in the literature, indicating a higher prevalence in middle-aged patients (30–50 years), or older.^[12]^ Other studies evaluating pain and QOL amongst patients with FMS consisted patients with a mean age of 46 and 50 years, respectively, in their samples.^[12]^ An international study in America found that 91% of their sample were of Caucasian ethnicity.^[13]^ This is similar to the findings in this research study, where the majority (68%) of the sample was Caucasian. In contrast, another international study concluded that individuals from African descent were 1.52 times more likely to be diagnosed with FMS in comparison to their Caucasian counterparts.^[14]^ Therefore, definite conclusions with regards to the influence of ethnicity on FMS cannot be shown. In this study, 61% of participants were employed either on a full-time or part-time basis, or were self-employed. Although 16% were unable to work, it was unclear whether this was owing to their FMS, as reasons for unemployment was not assessed in this questionnaire. In a study conducted in 2021, the majority of the sample (52%) were employed while living with FMS, although 6% were on long-term sick leave due to their FMS symptoms.^[12]^

### The impact of FMS on daily life

The overall impact of FMS on the life of the participants in this study was high (67±16%). Symptoms experienced had a large impact on daily life, while pain and daily function had a smaller impact. Previous studies reported similar results.^[15]^ Owing to the subjective nature of the questionnaires, it is evident that the participants in this study deemed their symptoms to affect their daily lives more than their physical limitations and pain. In addition, a recent study reported that pain is not the major predictor and contributor to quality of life amongst FMS patients.^[16]^ This notion is supported by previous research, which determined that tiredness and a depressed mood affected FMS participants more than their pain levels experienced.^[17]^ In a study examining the experiences of South African FMS patients, one participant reported; “The symptoms really started to become quite difficult … when I used to go to doctors saying… because at that point I was still physically very active … ‘this fatigue is unmanageable. I can’t cope with the fatigue’.”^[18]^ Another study reported that poor sleep quality and sleep disturbances were the most frequently reported complaints.^[12]^ This could not only be linked to fatigue, but by identifying subgroups of patients with similar experiences and symptoms, healthcare practitioners can improve the diagnostic criteria and treatment of the FMS.^[12]^

### Quality of life

The low overall QOL (<50%) in this study correlates with previous research showing that the QOL of FMS individuals is extremely low.^[19]^ The participants in this research study had low scores for vitality and pain. In addition, the same participants who reported low scores for vitality and pain, also required attention in the role limitations due to physical health and emotional problems. Similarly, a study done outside of South Africa indicated that participants yielded low scores in the mental health, social functioning, vitality, pain and general health categories.^[19]^ In another study, the areas with the lowest QOL scores included depression, general activity, general health perception, and vitality.^[16]^ It has been reported, however, that depression is a strong predictor of QOL and that depression and anxiety often mediate the effect of pain on overall QOL.^[20]^

### Physical activity, sedentary time and FMS

Contrary to previous research, 82% of participants in this study stated that they participated in some form of physical activity. In other research, the majority of individuals with FMS do not typically participate in physical activity and are classified as aerobically unfit. ^[21]^ Participants in this study reported that they spent a higher than usual amount of time engaging in some form of physical activity during the week. This included activity as part of work, travel to and from work, as well as recreational physical activity. It is possible that the participants were not fully able to differentiate between the different intensities of activity, leading to unusually high activity times shown. This could be reflected in the large amount of time spent being sedentary per day. High sedentary time has been associated with worse pain regulation, overall pain, fatigue, and disease impact in FMS patients.^[9]^ It has also been determined that substituting 30 minutes of sedentary time with light physical activity yielded better scores in the bodily pain, vitality and the social functioning domains of the SF-36, while all domains of the FIQR showed improvements. ^[9]^ Although sedentary time has been linked to increased pain, it has been postulated that pain does not play a role in physical activity participation amongst these patients. While research has shown that 60% to 80% of FMS patients believe physical activity to be beneficial for weight loss, fitness, and feelings of wellbeing, they reported that it was ineffective in reducing pain.^[22]^ The perception of its ineffectiveness in reducing pain could be a barrier to the increase in physical activity, or the patient’s willingness to continue to adhere to an exercise programme.

Previous research has shown a correlation between low sedentary time and high activity time to improved symptomology in FMS patients, as well as higher QOL.^[15,7]^ Due to the nature of the pilot study and the small sample size, the results of this study are inconclusive. However, it has been postulated that better QOL was common amongst those patients whose adherence to treatment is higher.^[12]^ For this reason, the researchers believe that if this study is replicated amongst a larger sample, findings would illustrate that higher physical activity levels and reduced sedentary time are linked to improved QOL and lower FMS impact.

### The relationship between QOL and impact of FMS

The last objective of this study was to determine the relationship between FMS and QOL. Findings illustrated that a strong correlation exists between FIQR and SF-36 in all domains. High FIQR scores were associated with low scores on the SF-36, indicating that the high impact of FMS experienced by the sample in daily life points toward a lower the QOL for the participant. This was especially true in the relationship to pain, where high pain levels resulted in poorer QOL. Pain in FMS is treated with pharmacology and physical therapy.^[23]^ While it has been said that some medications produce side effects severe enough to result in patients discontinuing, adherence to physical therapy, although beneficial, is also low due to pain tolerance and the willingness to participate. ^[23]^ Management of the condition should include a multidisciplinary approach, by integrating complementary medicine and non-pharmacological interventions to reduce pain, increase physical activity and improve QOL. ^[23]^ A study conducted in France also reported that their sample reported high impact scores and low QOL scores. ^[12]^ Their sample had shown deterioration in functional autonomy, increased severity of symptoms and emotional distress, as well as poor overall QOL. ^[12]^ This led to the researchers advocating for increased awareness and a call to healthcare practitioners to pay special attention to FMS patients, as a large majority tend to distrust the healthcare system, resulting in poorer adherence to medical treatment. ^[12]^

### Limitations

The generalisability of the findings in this study should be considered owing to the sample being recruited from the Johannesburg region only.

Ethnicities in the demographic section of the questionnaire were not applicable to the South African context, which could have made it challenging for participants to place themselves in this section.

The different intensities used in the GPAQ were not clearly defined. Participants may not have been able to clearly differentiate between the different intensities, leading to inaccurate recording of weekly activities.

Whether the participants were taking medications or receiving any form of treatment for their FMS was not assessed, which could have a significant impact on results of this study.

The COVID-19 pandemic and the South African lockdown resulted in the questionnaire being placed online and prevented its physical administration, which may have had an impact on the sample size.

This study did not assess the past and current treatment plans of the participants and how they might have influenced the results. It is possible that the participants are using only exercise as a treatment for the condition, as opposed to a multidisciplinary and holistic approach. This could possibly explain the positive relationship between QOL and exercise in the present study.

The body mass index was not assessed in this study, which could play a role in the FIQR and QOL scores. It is thus possible that the majority of participants in this study are overweight, which would influence the results.

## Conclusion

The findings in this study have demonstrated a large impact of daily life in South African FMS patients. It is also evident that these individuals have a low QOL, although participation in physical activity is evident. However, the link between exercise and QOL of these individuals is unclear. It is suggested that this study be repeated with a larger sample size to determine a clear link between physical activity and QOL amongst patients with FMS in South Africa.

## Supplementary Information



## Figures and Tables

**Table 1:** Demographic data of the participants (n=38)

Domain		Frequency (%)
**Gender**	Female	37 (97.4)
	Male	1 (2.6)

**Age**	18–20	1 (2.6)
	21–30	4 (10.5)
	31–40	6 (15.8)
	41–50	12 (31.6)
	51–60	11 (29.0)
	>60	4 (10.5)

**Ethnicity**	Caucasian	26 (68.5)
	Asian / Pacific Islander	4 (10.5)
	Mixed Ethnicity	4 (10.5)
	Indian	4 (10.5)
	Black African	0 (0.0)

**Employment status**	Employed full time	16 (42.0)
	Unable to work	6 (15.8)
	Unemployed but not currently looking for work	5 (13.2)
	Self-employed	5 (13.2)
	Employed part time	2 (5.3)
	Retired	2 (5.3)
	Unemployed and looking for work	1 (2.6)
	Student	1 (2.6)

**Table 2:** Impact of FMS on daily life (FIQR) (n=38)

Domain	Score (%)
Daily function	18.4 ± 5.7
Pain levels	13.4 ± 4.4
Symptoms experienced	35.5 ± 7.2
Total FIQR score	67.4 ± 16.1

Data are expressed as mean ± SD. The higher the score, the larger the impact on the participants life. FIQR, Fibromyalgia Impact Questionnaire – Revised; FMS, Fibromyalgia Syndrome.

**Table 3:** Quality of life of participants (SF-36) (n=38)

Domain	Score (%)
Physical functioning	34.9 ± 21.9
Role physical	15.1 ± 25.0
Role emotional	22.8 ± 33.9
Energy/Fatigue	22.2 ± 14.4
Emotional wellbeing	44.8 ± 18.4
Social functioning	39.8 ± 23.8
Pain	30.9 ± 20.9
General health	32.6 ± 18.8

Data are expressed as mean ± SD.

**Table 4:** Correlation between FIQR and QOL (n=38)

Domain	Pearson correlation (95% CI)
Physical functioning	−0.69 (−0.82, −0.46)
Role physical	−0.53 (−0.73, −0.24)
Role emotional	−0.51 (−0.71, −0.22)
Energy/Fatigue	−0.71 (−0.84, −0.50)
Emotional wellbeing	−0.72 (−0.84, −0.52)
Social functioning	−0.70 (−0.84, −0.49)
Pain	−0.71 (−0.84, −0.50)
General health	−0.55 (−0.74, −0.27)

All data shown are significant at the level of p <0.01 (after Bonferroni Adjustment). FIQR, Fibromyalgia Impact Questionnaire – Revised; QOL, quality of life

**Table 5:** Correlation between total active time, sedentary time and QOL (n=38)

	Total active time		Sedentary time	
	
Domain	Pearson Correlation (95% CI)	p value	Pearson Correlation (95% CI)	p value
Physical functioning	0.39 (0.08, 0.63)	0.016	0.09 (−0.24, 0.40)	0.604
Role physical	0.19 (−0.14, 0.48)	0.266	0.19 (−0.14, 0.48)	0.266
Role emotional	0.05 (−0.28, 0.36)	0.782	0.03 (−0.29, 0.35)	0.852
Energy/Fatigue	0.33 (0.01, 0.58)	0.047	0.09 (−0.23, 0.40)	0.580
Emotional wellbeing	0.16 (−0.17, 0.45)	0.350	0.11 (−0.22, 0.41)	0.523
Social functioning	0.05 (−0.28, 0.36)	0.774	0.25 (−0.08, 0.53)	0.130
Pain	0.13 (−0.19, 0.44)	0.422	0.01 (−0.32, 0.33)	0.974
General health	0.29 (−0.03, 0.56)	0.079	0.01 (−0.33, 0.32)	0.973

QOL, quality of life.
