# Supplementary material for: Physical activity and quality of life of patients with fibromyalgia
Source: S Afr J Sports Med. 2023 Feb 15;35(1):v35i1a14781. doi: 10.17159/2078-516X/2023/v35i1a14781 (PMC10798615; doi:10.17159/2078-516X/2023/v35i1a14781)
Supplement: Supplementary file 1 [file 2078-516X-35-v35i1a14781-s001.pdf]

Global Physical Activity Questionnaire (GPAQ)

WHO STEPwise approach to NCD risk factor surveillance

Surveillance and Population-Based Prevention
Prevention of Noncommunicable Diseases Department
World Health Organization
20 Avenue Appia, 1211 Geneva 27, Switzerland
For further information: www.who.int/chp/steps

GPAQ

Physical Activity

Next I am going to ask you about the time you spend doing different types of physical activity in a typical week. Please answer these questions even if you do not consider yourself to be a physically active person.

Think first about the time you spend doing work. Think of work as the things that you have to do such as paid or unpaid work, study/training, household chores, harvesting food/crops, fishing or hunting for food, seeking employment. *[Insert other examples if needed].* In answering the following questions 'vigorous-intensity activities' are activities that require hard physical effort and cause large increases in breathing or heart rate, 'moderate-intensity activities' are activities that require moderate physical effort and cause small increases in breathing or heart rate.

	Questions	Response	Code
**Activity at work**			
1	Does your work involve vigorous-intensity activity that causes large increases in breathing or heart rate like *[carrying or lifting heavy loads, digging or construction work*] for at least 10 minutes continuously? *[INSERT EXAMPLES] (USE SHOWCARD)*	Yes 1<br>No 2 *If No, go to P 4*	P1
2	In a typical week, on how many days do you do vigorous-intensity activities as part of your work?	Number of days └─┘	P2
3	How much time do you spend doing vigorous-intensity activities at work on a typical day?	Hours : minutes └─┴─┘ : └─┴─┘ hrs mins	P3 (a-b)
4	Does your work involve moderate-intensity activity that causes small increases in breathing or heart rate such as brisk walking *[or carrying light loads*] for at least 10 minutes continuously? *[INSERT EXAMPLES] (USE SHOWCARD)*	Yes 1<br>No 2 *If No, go to P 7*	P4
5	In a typical week, on how many days do you do moderate-intensity activities as part of your work?	Number of days └─┘	P5
6	How much time do you spend doing moderate-intensity activities at work on a typical day?	Hours : minutes └─┴─┘ : └─┴─┘ hrs mins	P6 (a-b)
**Travel to and from places**			
The next questions exclude the physical activities at work that you have already mentioned.<br>Now I would like to ask you about the usual way you travel to and from places. For example to work, for shopping, to market, to place of worship. [insert other examples if needed]			
7	Do you walk or use a bicycle (*pedal cycle*) for at least 10 minutes continuously to get to and from places?	Yes 1<br>No 2 *If No, go to P 10*	P7
8	In a typical week, on how many days do you walk or bicycle for at least 10 minutes continuously to get to and from places?	Number of days └─┘	P8
9	How much time do you spend walking or bicycling for travel on a typical day?	Hours : minutes └─┴─┘ : └─┴─┘ hrs mins	P9 (a-b)
**Recreational activities**			
The next questions exclude the work and transport activities that you have already mentioned.<br>Now I would like to ask you about sports, fitness and recreational activities (leisure), [insert relevant terms].			
10	Do you do any vigorous-intensity sports, fitness or recreational (*leisure*) activities that cause large increases in breathing or heart rate like [*running or football,]* for at least 10 minutes continuously? *[INSERT EXAMPLES] (USE SHOWCARD)*	Yes 1<br>No 2 *If No, go to P 13*	P10
11	In a typical week, on how many days do you do vigorous-intensity sports, fitness or recreational (*leisure*) activities?	Number of days └─┘	P11
12	How much time do you spend doing vigorous-intensity sports, fitness or recreational activities on a typical day?	Hours : minutes └─┴─┘ : └─┴─┘ hrs mins	P12 (a-b)

Continued on next page

**Physical Activity (recreational activities)** contd.			
**Questions**		**Response**	**Code**
13	Do you do any moderate-intensity sports, fitness or recreational *(leisure)* activities that causes a small increase in breathing or heart rate such as brisk walking,(*cycling, swimming, volleyball*)for at least 10 minutes continuously?<br>*[INSERT EXAMPLES] (USE SHOWCARD)*	Yes 1<br>No 2 *If No, go to P16*	P13
14	In a typical week, on how many days do you do moderate-intensity sports, fitness or recreational (*leisure*) activities?	Number of days └─┘	P14
15	How much time do you spend doing moderate-intensity sports, fitness or recreational (*leisure*) activities on a typical day?	Hours : minutes └─┴─┘ : └─┴─┘<br>hrs mins	P15 (a-b)
**Sedentary behaviour**			
The following question is about sitting or reclining at work, at home, getting to and from places, or with friends including time spent [sitting at a desk, sitting with friends, travelling in car, bus, train, reading, playing cards or watching television], but do not include time spent sleeping.<br>*[INSERT EXAMPLES] (USE SHOWCARD)*			
16	How much time do you usually spend sitting or reclining on a typical day?	Hours : minutes └─┴─┘ : └─┴─┘<br>hrs min s	P16 (a-b)

SF-36 QUESTIONNAIRE

Name:____________________ **Ref. Dr:___________________** **Date: _______**

ID#: _______________ **Age: _______** **Gender: M / F**

Please answer the 36 questions of the **Health Survey** completely, honestly, and without interruptions.

GENERAL HEALTH:
In general, would you say your health is:
◯ Excellent ◯ Very Good ◯ Good ◯ Fair ◯ Poor

Compared to one year ago, how would you rate your health in general now?
◯ Much better now than one year ago
◯ Somewhat better now than one year ago
◯ About the same
◯ Somewhat worse now than one year ago
◯ Much worse than one year ago

LIMITATIONS OF ACTIVITIES:
The following items are about activities you might do during a typical day. Does your health now limit you in these activities? If so, how much?

Vigorous activities, such as running, lifting heavy objects, participating in strenuous sports.
◯ Yes, Limited a lot ◯ Yes, Limited a Little ◯ No, Not Limited at all

Moderate activities, such as moving a table, pushing a vacuum cleaner, bowling, or playing golf
◯ Yes, Limited a Lot ◯ Yes, Limited a Little ◯ No, Not Limited at all

Lifting or carrying groceries
◯ Yes, Limited a Lot ◯ Yes, Limited a Little ◯ No, Not Limited at all

Climbing several flights of stairs
◯ Yes, Limited a Lot ◯ Yes, Limited a Little ◯ No, Not Limited at all

Climbing one flight of stairs
◯ Yes, Limited a Lot ◯ Yes, Limited a Little ◯ No, Not Limited at all

Bending, kneeling, or stooping
◯ Yes, Limited a Lot ◯ Yes, Limited a Little ◯ No, Not Limited at all

Walking more than a mile
◯ Yes, Limited a Lot ◯ Yes, Limited a Little ◯ No, Not Limited at all

Walking several blocks
◯ Yes, Limited a Lot ◯ Yes, Limited a Little ◯ No, Not Limited at all

Walking one block
◯ Yes, Limited a Lot ◯ Yes, Limited a Little ◯ No, Not Limited at all

Bathing or dressing yourself

◯ Yes, Limited a Lot ◯ Yes, Limited a Little ◯ No, Not Limited at all

PHYSICAL HEALTH PROBLEMS:

During the past 4 weeks, have you had any of the following problems with your work or other regular daily activities as a result of your physical health?

Cut down the amount of time you spent on work or other activities

◯ Yes ◯ No

Accomplished less than you would like

◯ Yes ◯ No

Were limited in the kind of work or other activities

◯ Yes ◯ No

Had difficulty performing the work or other activities (for example, it took extra effort)

◯ Yes ◯ No

EMOTIONAL HEALTH PROBLEMS:

During the past 4 weeks, have you had any of the following problems with your work or other regular daily activities as a result of any emotional problems (such as feeling depressed or anxious)?

Cut down the amount of time you spent on work or other activities

◯ Yes ◯ No

Accomplished less than you would like

◯ Yes ◯ No

Didn't do work or other activities as carefully as usual

◯ Yes ◯ No

SOCIAL ACTIVITIES:

Emotional problems interfered with your normal social activities with family, friends, neighbors, or groups?

◯ Not at all ◯ Slightly ◯ Moderately ◯ Severe ◯ Very Severe

PAIN:

How much bodily pain have you had during the past 4 weeks?

◯ None ◯ Very Mild ◯ Mild ◯ Moderate ◯ Severe ◯ Very Severe

During the past 4 weeks, how much did pain interfere with your normal work (including both work outside the home and housework)?

◯ Not at all ◯ A little bit ◯ Moderately ◯ Quite a bit ◯ Extremely

ENERGY AND EMOTIONS:
These questions are about how you feel and how things have been with you during the last 4 weeks. For each question, please give the answer that comes closest to the way you have been feeling.

Did you feel full of pep?
- All of the time
- Most of the time
- A good Bit of the Time
- Some of the time
- A little bit of the time
- None of the Time

Have you been a very nervous person?
- All of the time
- Most of the time
- A good Bit of the Time
- Some of the time
- A little bit of the time
- None of the Time

Have you felt so down in the dumps that nothing could cheer you up?
- All of the time
- Most of the time
- A good Bit of the Time
- Some of the time
- A little bit of the time
- None of the Time

Have you felt calm and peaceful?
- All of the time
- Most of the time
- A good Bit of the Time
- Some of the time
- A little bit of the time
- None of the Time

Did you have a lot of energy?
- All of the time
- Most of the time
- A good Bit of the Time
- Some of the time
- A little bit of the time
- None of the Time

Have you felt downhearted and blue?

- All of the time
- Most of the time
- A good Bit of the Time
- Some of the time
- A little bit of the time
- None of the Time

Did you feel worn out?

- All of the time
- Most of the time
- A good Bit of the Time
- Some of the time
- A little bit of the time
- None of the Time

Have you been a happy person?

- All of the time
- Most of the time
- A good Bit of the Time
- Some of the time
- A little bit of the time
- None of the Time

Did you feel tired?

- All of the time
- Most of the time
- A good Bit of the Time
- Some of the time
- A little bit of the time
- None of the Time

SOCIAL ACTIVITIES:
During the past 4 weeks, how much of the time has your physical health or emotional problems interfered with your social activities (like visiting with friends, relatives, etc.)?

- All of the time
- Most of the time
- Some of the time
- A little bit of the time
- None of the Time

GENERAL HEALTH:
How true or false is each of the following statements for you?

I seem to get sick a little easier than other people
○ Definitely true ○ Mostly true ○ Don't know ○ Mostly false ○ Definitely false

I am as healthy as anybody I know
○ Definitely true ○ Mostly true ○ Don't know ○ Mostly false ○ Definitely false

I expect my health to get worse
○ Definitely true ○ Mostly true ○ Don't know ○ Mostly false ○ Definitely false

My health is excellent
○ Definitely true ○ Mostly true ○ Don't know ○ Mostly false ○ Definitely false

REVISED FIBROMYALGIA
IMPACT QUESTIONNAIRE (FIQR)

Last Name: ______________________ **Duration of FM symptoms (years):** __________

First Name: ______________________ **Time since FM was first diagnosed (years):** __________

Age: ______________________

DOMAIN 1: FUNCTION

Directions: For each of the following 9 questions, check the box that best indicates how much your Fibromyalgia made it difficult to perform each of the following activities during the past 7 days. If you did not perform a particular activity in the last 7 days, rate the difficulty for the last time you performed the activity. If you can't perform an activity, check the last box.

BRUSH OR COMB YOUR HAIR

**No difficulty**	❑ 0	❑ 1	❑ 2	❑ 3	❑ 4	❑ 5	❑ 6	❑ 7	❑ 8	❑ 9	❑ 10	**Very difficult**

WALK CONTINUOUSLY FOR 20 MINUTES

**No difficulty**	❑ 0	❑ 1	❑ 2	❑ 3	❑ 4	❑ 5	❑ 6	❑ 7	❑ 8	❑ 9	❑ 10	**Very difficult**

PREPARE A HOMEMADE MEAL

**No difficulty**	❑ 0	❑ 1	❑ 2	❑ 3	❑ 4	❑ 5	❑ 6	❑ 7	❑ 8	❑ 9	❑ 10	**Very difficult**

VACUUM, SCRUB, OR SWEEP FLOORS

**No difficulty**	❑ 0	❑ 1	❑ 2	❑ 3	❑ 4	❑ 5	❑ 6	❑ 7	❑ 8	❑ 9	❑ 10	**Very difficult**

LIFT AND CARRY A BAG FULL OF GROCERIES

**No difficulty**	❑ 0	❑ 1	❑ 2	❑ 3	❑ 4	❑ 5	❑ 6	❑ 7	❑ 8	❑ 9	❑ 10	**Very difficult**

CLIMB ONE FLIGHT OF STAIRS

**No difficulty**	❑ 0	❑ 1	❑ 2	❑ 3	❑ 4	❑ 5	❑ 6	❑ 7	❑ 8	❑ 9	❑ 10	**Very difficult**

CHANGE BEDSHEETS

**No difficulty**	❑ 0	❑ 1	❑ 2	❑ 3	❑ 4	❑ 5	❑ 6	❑ 7	❑ 8	❑ 9	❑ 10	**Very difficult**

SIT IN A CHAIR FOR 45 MINUTES

**No difficulty**	❑ 0	❑ 1	❑ 2	❑ 3	❑ 4	❑ 5	❑ 6	❑ 7	❑ 8	❑ 9	❑ 10	**Very difficult**

SHOP FOR GROCERIES

	❑	❑	❑	❑	❑	❑	❑	❑	❑	❑	❑	
**No difficulty**	0	1	2	3	4	5	6	7	8	9	10	**Very difficult**

DOMAIN 1 SUBTOTAL:_______________

DOMAIN 2: OVERALL

Directions: For each of the following 2 questions, check the box that best describes the overall impact of your
Fibromyalgia over the last 7 days.

FIBROMYALGIA PREVENTED ME FROM ACCOMPLISHING GOALS FOR THE WEEK

	❑	❑	❑	❑	❑	❑	❑	❑	❑	❑	❑	
**Never**	0	1	2	3	4	5	6	7	8	9	10	**Always**

I WAS COMPLETELY OVERWHELMED BY MY FIBROMYALGIA SYMPTOMS

	❑	❑	❑	❑	❑	❑	❑	❑	❑	❑	❑	
**Never**	0	1	2	3	4	5	6	7	8	9	10	**Always**

DOMAIN 2 SUBTOTAL:_______________

DOMAIN 3: SYMPTOMS

Directions: For each of the following 10 questions, select the box that best indicates your intensity level of these common
Fibromyalgia symptoms over the past 7 days.

PLEASE RATE THE LEVEL OF PAIN

	❑	❑	❑	❑	❑	❑	❑	❑	❑	❑	❑	
**No pain**	0	1	2	3	4	5	6	7	8	9	10	**Unbearable pain**

PLEASE RATE YOUR LEVEL OF ENERGY

	❑	❑	❑	❑	❑	❑	❑	❑	❑	❑	❑	
**Lots of energy**	0	1	2	3	4	5	6	7	8	9	10	**No energy**

PLEASE RATE YOUR LEVEL OF STIFFNESS

	❑	❑	❑	❑	❑	❑	❑	❑	❑	❑	❑	
**No stiffness**	0	1	2	3	4	5	6	7	8	9	10	**Severe stiffness**

PLEASE RATE THE QUALITY OF YOUR SLEEP

	❑	❑	❑	❑	❑	❑	❑	❑	❑	❑	❑	
**Awoke well rested**	0	1	2	3	4	5	6	7	8	9	10	**Awoke very tired**

PLEASE RATE YOUR LEVEL OF DEPRESSION

**No depression**	❑ 0	❑ 1	❑ 2	❑ 3	❑ 4	❑ 5	❑ 6	❑ 7	❑ 8	❑ 9	❑ 10	**Very depressed**

PLEASE RATE YOUR LEVEL OF MEMORY PROBLEMS

**Good memory**	❑ 0	❑ 1	❑ 2	❑ 3	❑ 4	❑ 5	❑ 6	❑ 7	❑ 8	❑ 9	❑ 10	**Very poor memory**

PLEASE RATE YOUR LEVEL OF ANXIETY

**Not anxious**	❑ 0	❑ 1	❑ 2	❑ 3	❑ 4	❑ 5	❑ 6	❑ 7	❑ 8	❑ 9	❑ 10	**Very anxious**

PLEASE RATE YOUR LEVEL OF TENDERNESS TO TOUCH

**No tenderness**	❑ 0	❑ 1	❑ 2	❑ 3	❑ 4	❑ 5	❑ 6	❑ 7	❑ 8	❑ 9	❑ 10	**Very tender**

PLEASE RATE YOUR LEVEL OF BALANCE PROBLEMS

**No imbalance**	❑ 0	❑ 1	❑ 2	❑ 3	❑ 4	❑ 5	❑ 6	❑ 7	❑ 8	❑ 9	❑ 10	**Severe imbalance**

PLEASE RATE YOUR LEVEL OF SENSITIVITY TO LOUD NOISES, BRIGHT LIGHTS, ODORS, AND COLD

**No sensitivity**	❑ 0	❑ 1	❑ 2	❑ 3	❑ 4	❑ 5	❑ 6	❑ 7	❑ 8	❑ 9	❑ 10	**Extreme sensitivity**

DOMAIN 3 SUBTOTAL:_______________

SCORING:

1) Sum the scores for each of the 3 domains (function, overall, and symptoms)

2) Divide domain 1 score by 3, leave domain 2 score unchanged, and divide domain 3 score by 2

3) Add the 3 resulting domain scores to obtain the total FIQR score

**DOMAIN 1 SUBTOTAL**	____________	**÷ 3**	=	____________	
**DOMAIN 2 SUBTOTAL**	____________	**CARRY OVER SUBTOTAL**	=	____________	**TOTAL FIQR SCORE**
**DOMAIN 3 SUBTOTAL**	____________	**÷ 2**	=	____________	

10% TOTAL RECOVERED FIBER

PBP01671B1/PBP420315-01

© 2012 Pfizer Inc. All rights reserved.

Printed in USA/February 2012

Pfizer Working together for a healthier world™
